# Single-molecule FRET studies on alpha-synuclein oligomerization of Parkinson’s disease genetically related mutants

**DOI:** 10.1038/srep16696

**Published:** 2015-11-19

**Authors:** Laura Tosatto, Mathew H. Horrocks, Alexander J. Dear, Tuomas P. J. Knowles, Mauro Dalla Serra, Nunilo Cremades, Christopher M. Dobson, David Klenerman

**Affiliations:** 1 Department of Chemistry, University of Cambridge, Lensfield Road, CB2 1EW Cambridge, UK; 2 Istituto di Biofisica, Consiglio Nazionale delle Ricerche, via alla Cascata 56/C, 38123 Trento, Italy; 3 Institute for Biocomputation and Physics of Complex Systems (BIFI), Universidad de Zaragoza, Mariano Esquillor, Edificio I+D, 50018 Zaragoza, Spain

## Abstract

Oligomers of alpha-synuclein are toxic to cells and have been proposed to play a key role in the etiopathogenesis of Parkinson’s disease. As certain missense mutations in the gene encoding for alpha-synuclein induce early-onset forms of the disease, it has been suggested that these variants might have an inherent tendency to produce high concentrations of oligomers during aggregation, although a direct experimental evidence for this is still missing. We used single-molecule Förster Resonance Energy Transfer to visualize directly the protein self-assembly process by wild-type alpha-synuclein and A53T, A30P and E46K mutants and to compare the structural properties of the ensemble of oligomers generated. We found that the kinetics of oligomer formation correlates with the natural tendency of each variant to acquire beta-sheet structure. Moreover, A53T and A30P showed significant differences in the averaged FRET efficiency of one of the two types of oligomers formed compared to the wild-type oligomers, indicating possible structural variety among the ensemble of species generated. Importantly, we found similar concentrations of oligomers during the lag-phase of the aggregation of wild-type and mutated alpha-synuclein, suggesting that the properties of the ensemble of oligomers generated during self-assembly might be more relevant than their absolute concentration for triggering neurodegeneration.

Parkinson’s disease (PD) is caused by the selective loss of specific dopaminergic neurons involved in the generation and coordination of movements^1^. The molecular mechanisms leading to neuronal death are still unknown; however alpha-synuclein (aS) is known to play a central role. Amyloid-like fibrils of this intrinsically disordered protein are found in the *substantia nigra pars compacta* of the brains of PD patients^2^,^3^,^4^; furthermore, six point mutations and the gene duplication and triplication itself cause autosomal dominant early onset forms of the disease^5^,^6^,^7^,^8^,^9^,^10^,^11^,^12^. It is interesting to notice that the pathological mutations are located in a region of aS that is not necessary for fibril formation^13^, although their presence strongly influences the aggregation propensities of aS: some mutations increase the aggregation rate of the protein while others induce a significant reduction in the rate of fibril formation^14^,^15^,^16^,^17^,^18^. In particular, the A30P mutation results in an increased lag-phase for fibril formation. This effect has been attributed to a decreased ability of the N-terminal part of the protein to acquire secondary structure by the introduction of a Proline residue at position 30, which in turn would result in the destabilization of beta-sheet rich aggregates whose formation involve the folding of this protein region, and, therefore, in a lower tendency to form amyloid fibrils^16^,^19^,^20^,^21^,^22^,^23^. NMR and molecular dynamics simulations studies of A53T variant suggest that the protein presents an increased propensity to acquire beta-sheet structure when the Alanine residue at position 53 is mutated to Threonine^19^,^24^,^25^,^26^,^27^; effect that has been correlated with the faster apparent aggregation rate of this mutant obtained experimentally^16^,^28^,^29^. A similar aggregation behaviour has been observed for the E46K mutation^15^,^30^, although in this case the decrease in the net charge of the protein, due to the charge swap resulting from the mutation, might be the cause of a higher propensity for aggregation^31^. Figure 1 summarizes the location in the protein sequence of the three missense mutations that we have studied here and their suggested effect on the ability of the protein to acquire secondary structure.

*In vitro* aS aggregation results in the formation of small soluble oligomers, which have been shown to be cytotoxic^32^,^33^,^34^, and associate further to form fibrils that are morphologically indistinguishable from the ones collected from post mortem brains^4^. AS aggregation occurs via a nucleation-dependent mechanism: the self-interaction needed for the formation of nuclei is thermodynamically un-favourable; once achieved, however, fibril elongation is triggered^35^. The characterization of the oligomeric species generated during the lag-phase before fibril formation has been demonstrated to be particularly challenging due to their intrinsic transient, heterogeneous and low abundant nature. Their identification and characterization during the aggregation reaction has been primarily derived from either indirect experimental data^16^,^28^,^34^ or by means of enriching the samples in certain types of highly stable oligomeric species^29^,^36^,^37^,^38^. By using these approaches, the pathological mutations have been reported to enhance the accumulation of oligomeric species during the aggregation reaction^16^,^28^,^29^. Other fluorescence-based techniques have been used to detect aS oligomers: fluorescence correlation spectroscopy has been applied to observe the fast generation of oligomers by aS and its pathological mutants in the presence of SDS^39^; to monitor the change in the hydrodynamic radius of oligomers with time^40^,^41^ or to follow the changes in the local environment of a sensitive probe such as pyrene^42^. Other techniques starting from highly concentrated solutions of aS to follow the evolution of aS aggregates with time, using AFM imaging together with FT-IR and complementary techniques, observed the formation of heterogeneous ensemble of species^43^,^44^,^45^. It would be desirable that direct information on the different types of oligomers formed during the aggregation of the protein could be obtained simultaneously in order to understand the differences between the aggregation pathways, the numbers and the structures of the oligomers generated when the wild-type (WT) protein and the pathological missense mutations aggregate, since this may provide new insights into the molecular mechanisms of aggregation for these mutants.

We have recently developed a novel single-molecule fluorescence approach that has allowed us to characterize in detail the oligomerization process of a number of amyloidogenic proteins and peptides^33^,^46^,^47^. In this method, an equimolar mixture of aS labelled either with AlexaFluor 488 or AlexaFluor 594 is incubated at 37 °C with shaking. Regular timepoints are taken during the aggregation reaction and are immediately diluted to picomolar concentrations to allow for detection on a custom-built single-molecule confocal microscope (Fig. 2A). During the aggregation reaction, oligomers are formed, and are likely to have both AlexaFluor 488 and AlexaFluor 594 labelled monomers. By observing molecules individually, it is possible to separate the signals generated from oligomeric species from an excess of monomer since oligomers give rise to a simultaneous burst of fluorescence intensity in both AlexaFluor 488 and AlexaFluor 594 channels (Fig. 2B). Furthermore, as AlexaFluor 488 and AlexaFluor 594 are a FRET pair, the oligomer fluorescence intensities can be used to estimate the oligomer size and determine the FRET efficiency of the oligomer (Figure 2B). Importantly, we have now incorporated fast-flow microfluidic techniques to the single-molecule fluorescence technique in order to increase the rate of data acquisition and ameliorate effects from the free diffusion of particles through the edges of the confocal volume^48^; this approach has been recently validated and their application has allowed us to obtained more detailed information of the nature of the different amyloid oligomeric species formed^49^. In the case of aS we were able to distinguish different structural groups of oligomers and determine the mechanism for their formation by using single-molecule intermolecular FRET (smFRET)^33^,^49^. Through this, we identified a key slow conversion step from the initially formed oligomers (referred as type-A oligomers), which were characterized by low FRET efficiency values, high sensitivities to proteinase K degradation and low or no toxicity, to more compact oligomers (type-B) that displayed higher FRET efficiency values, a higher resistance against proteolysis, presence of a beta-sheet folding core (ca. half of the beta-sheet content present in the fibrillar species^50^), and, importantly, highly cytotoxic properties^33^,^50^.

In this work, we have made use of this smFRET approach to characterize the oligomerization of three of the aS pathological mutants (A53T, A30P, E46K). Our results show that the fraction of oligomeric species generated during the primary nucleation of amyloid fibrils is similar when comparing the aS pathological variants with the WT protein. Importantly, our findings indicate that, in contrast to what has been widely thought, the pathological mutants do not have an inherent tendency to accumulate as oligomers, which has important consequences for developing our understanding of some genetic forms of PD. Moreover, E46K presented a different behaviour compared to the wild-type protein, giving rise to an oligomeric distribution in which the type-A and type-B forms could not be easily separated. Finally, the type-B oligomers formed from the A53T and A30P mutants had different FRET efficiencies to those formed from WT aS, indicating structural variations in the type-B oligomeric ensemble depending on the protein variant.

## Results

### Kinetic of formation of oligomers by WT* aS and mutants*

In order to characterize the oligomerization process of aS during the early stages of fibril formation in a rigorous manner we have to select carefully the time window at which the data would reflect primarily this process and thereby minimize the contribution of possible secondary processes in the reaction such as monomer consumption by fibril elongation and possible fibril surface-catalysed oligomerization^51^. As the different protein variants show different kinetics of aggregation, this time window varies depending on the protein variant (see Supp Info and Figure S2 for the analysis of the aggregation propensities and the selection of the experimental time window): 48 hours of aggregation for the WT* protein, 30 hours for A53T* and 96 hours for A30P* (* denotes fluorescently labelled protein).

**Figure 2:**
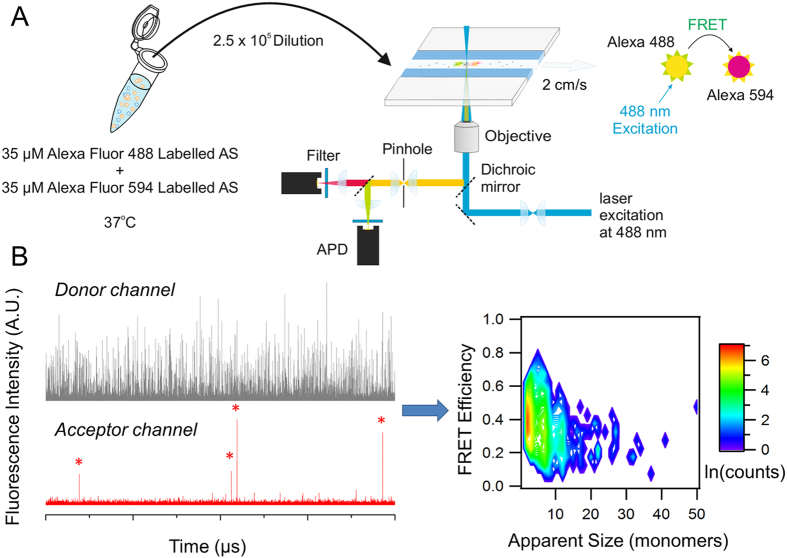
(**A**) Schematic of the single-molecule FRET experiments showing the instrumental set-up. An equimolar mixture of Alexa Fluor 488 and Alexa Fluor 594 labelled aS is incubated at 37 °C with 200 rpm shaking; aliquots are taken at regular time-points and diluted 1:250,000. The diluted solution is delivered to the probe volume of a confocal microscope equipped for single-molecule detection via a simple one-channelled microfluidic devices. The channel size, flow settings, and concentrations are chosen to provide discrete single-molecule fluorescence bursts^48^,^49^. (**B**) Example of fluorescent bursts and the FRET efficiency and apparent size analysis for each time-point: fluorescence bursts are detected when a molecule transits through the confocal volume; since only the 488 nm laser is used for excitation, bursts in the acceptor channel can only be detected when oligomers containing both donor and acceptor molecules pass through the confocal volume. Coincident signals from oligomers are collected and processed; the heterogeneity of the oligomers detected is shown in the 2D contour plot reporting the logarithm of the counts for each oligomer plotted in terms of FRET efficiency and apparent size. Typically, the number of events collected is between 100 and 1000.

**Figure 3:**
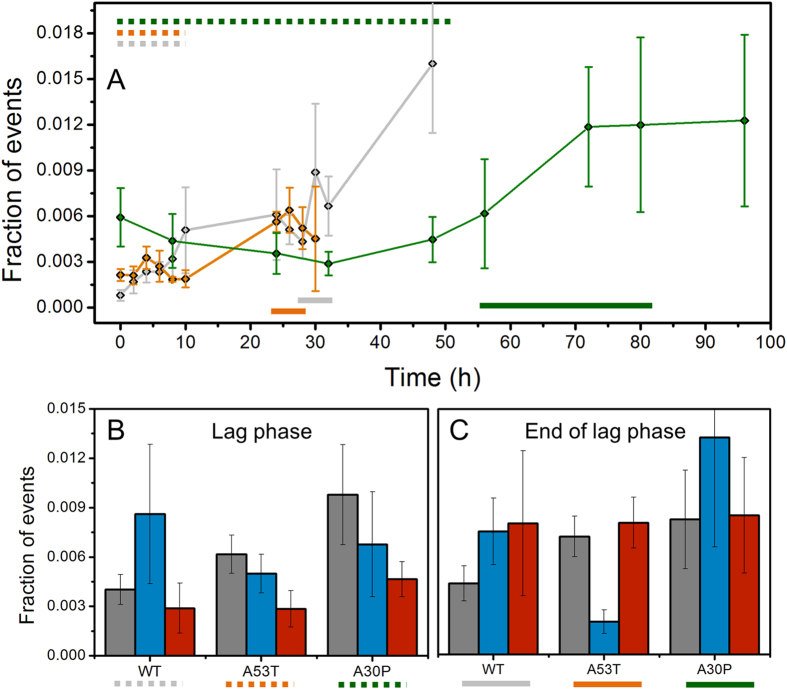
(**A**) Kinetic of formation of the total number of oligomers for WT* (grey; n = 3), A53T* (orange; n = 5) and A30P* (green; n = 6). The bars in the figure indicate the part of the kinetic considered for the calculation of fraction of events corresponding for the lag-phase (dotted) or end of the lag-phase (straight) part of the kinetic. (**B**) Histograms indicating the amount of apparent dimers (grey), type-A (blue) and type-B oligomers (red) in the time-points preceding fibrils formation during the lag phase or (**C**) at the end of the lag-phase. Error bars reported are the sum of SEM for each time-point added.

We set out to investigate protein oligomerization of each protein variant by taking small aliquots of the aggregation reaction at different time-points during the corresponding time window of analysis, diluting the aliquots to picomolar concentrations for single-molecule detection and performing smFRET analysis using a custom-built single-molecule fluorescence microscope^49^ (Fig. 2). Coincident fluorescent bursts in the green (emission of the FRET donor probe) and red (emission of the FRET acceptor probe) channels correspond to oligomers crossing the confocal volume, whereas events showing signal in the green channel only are indicative of monomers. Figure 3A shows the total number of oligomers generated during the aggregation of WT*, A53T* and A30P* aS. Interestingly, the total number of oligomers observed to accumulate during the lag-phase of the aggregation of the pathological mutants is in the same order of magnitude within error as that found for the WT* protein.

Our smFRET approach is able not only to determine the fraction of oligomeric species in a given sample, but also to obtain estimations of the size (deduced from fluorescence intensities) and structural group (deduced from FRET efficiencies) for each individual oligomeric species detected^33^. By sampling a large number of events per sample we can build two-dimensional contour plots that represent the ensemble of oligomers present at each time point during the aggregation reaction of the protein as a function of their apparent size and their FRET efficiency (Fig. 2B). At higher intensities, which can be attributed to larger apparent sizes of oligomers, two FRET populations of oligomers can be observed, one population centred at FRET efficiency values between 0.30 and 0.41, depending on the aS variant, and the other centred between 0.44 and 0.59. Each population was successfully fit to a Gaussian distribution, and was assigned to type-A and type-B oligomers, respectively^33^,^49^. At lower apparent sizes (detected as apparent dimers), and therefore lower intensities, the two populations could not be resolved, likely reflecting thresholding limitations, and they were analysed separately.

Figure 3B,C show the cumulative formation of apparent dimers, type-A and type-B oligomers during the initial time of the lag-phase (5 time-points: 1–10 hours for WT* and A53T*; till 48 hours for A30P*) and at the end of the lag-phase (3 time-points: 28–32 hours for the WT*, 24–28 hours for A53T*, 56–80 hours for A30P*). The percentage of oligomers for each protein variant has been reported in Table S1. For WT* aS, the majority of species detected in the lag-phase were type-A oligomers, whereas for A53T* and A30P*, the majority were apparent dimers followed by type-A oligomers. According to our previous results^33^,^49^, type-B oligomers are formed later and accumulate at the end of the lag-phase as result of a structural conversion of type-A oligomers^33^. Furthermore, the number of apparent dimers formed by A30P* is approximately double that formed by WT* protein, in a period of time 4 times longer, suggesting a delay in the growth/elongation of the apparent dimers rather than in their formation in the A30P variant. We can then compare the relative fractions of structural groups of oligomers at the end of the lag-phase to visualize further differences in the oligomerization process between the protein variants. A53T* variant show a remarkable (3-fold) reduction in the fraction of type-A oligomers in comparison to the WT* protein, suggesting a faster conversion of this group of oligomer to generate type-B oligomers, which are then converted to fibrils (likely at a similar rate as for the WT* protein). In contrast, the A30P* variant seems to accumulate type-A oligomers at the end of the lag-phase, indicative of a slower conversion from type-A to type-B oligomers. Figures S4 and S5 in the Supplementary Information show the kinetics of oligomer formation for WT* and mutants*.

### Oligomers conversion rates

In our previous works^33^,^49^, the smFRET data were fit to an model describing the aggregation process as a nucleation growth model with a conformational conversion step^52^; the same equation has been used here to compare the kinetic of oligomer formation and conversion for WT*, A53T* and A30P* aS. In this model, the primary nucleation arises from the generation of oligomers of type-A from monomeric aS, and type-A oligomers can grow through the addition of monomer or can convert into type-B oligomers, describing parallel pathways for the formation of type-B oligomers of a given size (details in the Supplementary Information section). Results indicated that oligomers formation rate (~5·10^−8^ s^−1^ for WT*, ~4·10^−8^ s^−1^ for A53T* and ~2·10^−8^ s^−1^ for A30P*) and conversion rate (~2·10^−5^ s^−1^ for WT*, ~5·10^−5^ s^−1^ for A53T* and ~6·10^−6^ s^−1^ for A30P*) are within the same order of magnitude, nevertheless reporting higher rates for oligomers conversion for A53T* compared to WT* and slower rates for A30P*.

### Analysis of E46K aS behaviour

**Figure 4:**
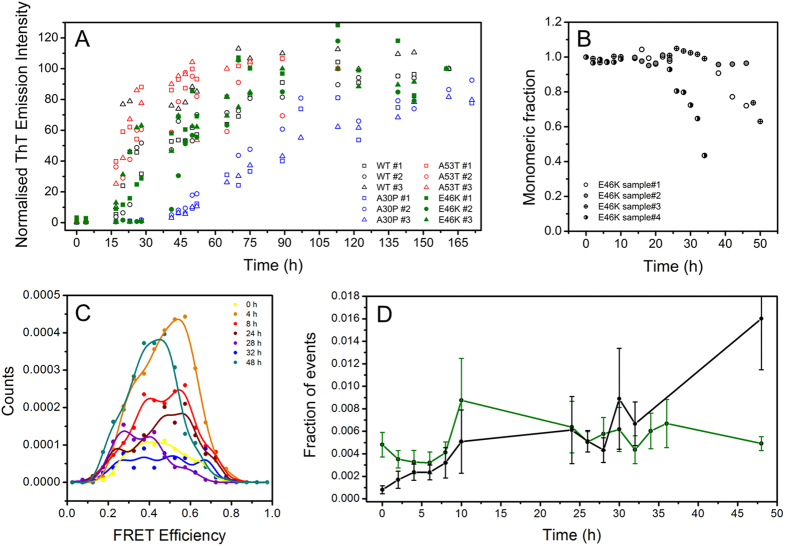
(**A**) Thioflavin T assay showing separate experiments of WT (black), A53T (red), A30P (blue) and E46K (green). Circles, triangles and squares represent three distinct experiments, for which the average in shown in Figure S1 for WT, A53T and A30P. To highlights E46K signals, green symbols are filled. Signals have been normalized after the achievement of the plateau. (**B**) Monomer concentration decay during the aggregation of 4 separate samples prepared from the same stock solution of E46K*. (**C**) Representative FRET efficiency distribution from one of the aggregation experiments of E46K* at 0h (yellow), 4 h (orange), 8 h (red), 24 h (brown), 28h (violet), 32 h (blue), 48 h (dark cyan). (**D**) SmFRET kinetic reporting the fraction of events for total oligomers content for E46K* (green; n = 7) and WT* (black; n = 3) aS; error bars reported are SEM.

For the E46K mutant, we found a large variation in the duration of the lag-phase for the individual experiments, compared to the WT protein or to the A53T and A30P mutants, both for the unlabelled (Fig. 4A) and the labelled protein (Fig. 4B). Labelled E46K (E46K*) lag-phases were observed to extend from 24 h to more than 48 h. Moreover, E46K* is a unique case, since it is not possible to separate clearly the type-A and B oligomer populations (one representative case is shown in Fig. 4C). Instead, a broad single distribution is observed (centred at 0.47 ± 0.04, with a width of 0.13 ± 0.01), and the total number of oligomers formed by E46K* is in the same order of magnitude of the total number of oligomers formed by WT* aS (Fig. 4D).

### Comparison of the FRET efficiencies distributions collected for WT* aS and mutants*

**Figure 5:**
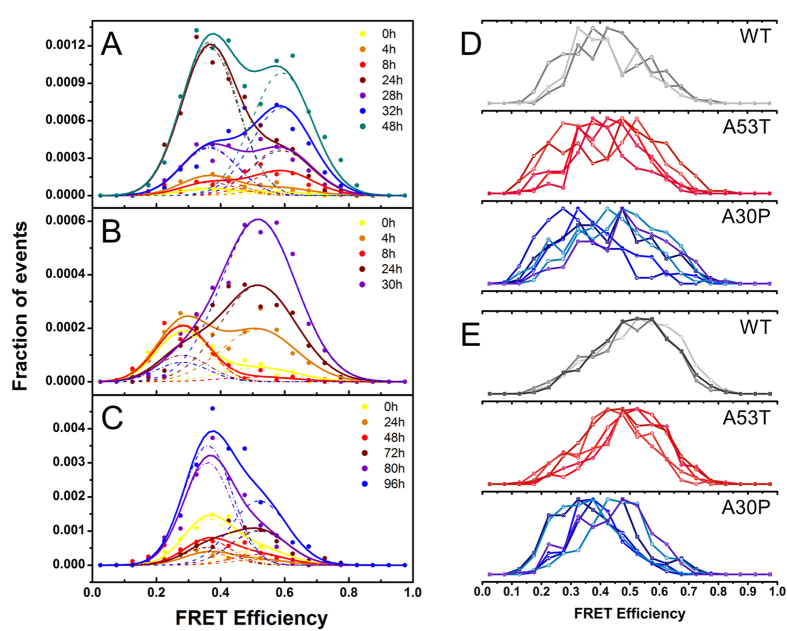
Example for Gaussian fits for FRET efficiency distribution from one of the aggregation kinetics of WT* or mutants using global fitting analysis. Dashed lines represent the contribution of type-A and type-B oligomers to the final fit (continuous line) for (**A**) WT*, (**B**) A53T* and (**C**) A30P*. (**D**) Oligomers FRET efficiencies distributions collected for early or late timepoints during the aggregation of WT aS and pathological variants. Different samples are indicated with different symbols and colour gradations; each curve is normalized to the highest number of oligomers to allow better comparison. Histograms of oligomers FRET efficiencies collected as early events at 4 hours for WT* (grey lines and symbols), 4 hours for A53T* (red lines and symbols) and 8 hours for A30P* (blue lines and symbols). (**E**) Histograms of oligomers FRET efficiencies collected at the end of the lag-phase at 32 hours for WT* (grey lines and symbols), 26 hours for A53T* (red lines and symbols) and 72 or 80 hours for A30P* (blue lines and symbols).

**Figure 6:**
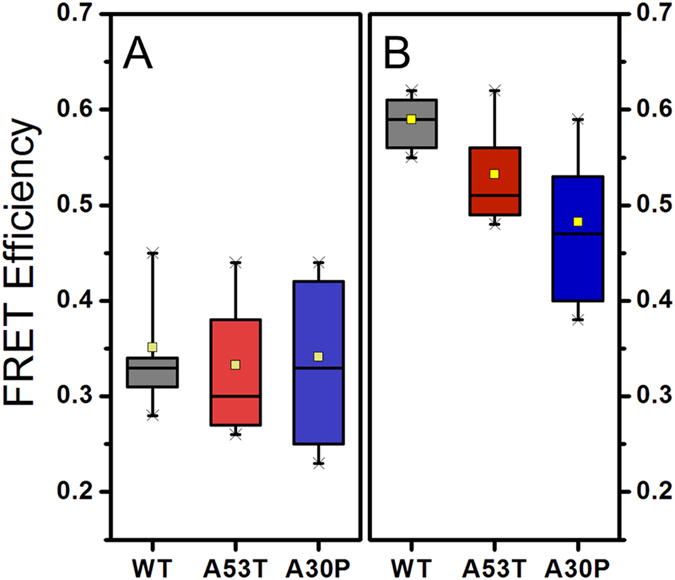
Box plot reporting values of the Gaussian curves centres obtained through global fitting of WT* (grey), A53T* (red) and A30P* (blue) FRET efficiency histograms. N = 8 for each protein variant. Right panel (**A**) reports values obtained for type-A oligomers, while left panel (**B**) reports values from type-B oligomers. Box boundaries represents 25 and 75 percentiles, the average is indicated with the yellow square, while whiskers represent 1 and 99 percentiles. Values of the fitted data are reported in Table S2 for each sample.

In order to determine whether there are differences in the structural organization of oligomers, we analysed the FRET efficiency values for the type-A and type-B oligomers for WT* and mutants* in detail. FRET efficiency is normally used to measure the distance between two fluorophores; however, in this case where there are multiple fluorophores present in one protein species, it can only be used as a way to identify different structural groups of oligomers and, given the position of the fluorophores and the structural information of that position in the aS fibrils, as a measure of the compactness of the oligomers^33^: if the oligomers are more compact and closer to the fibrillar structure (as was predicted for type-B oligomers^33^ and recently demonstrated^50^), then the dyes will be, on average, closer in distance, and, therefore, they would display higher FRET efficiency values. Figure 5A–C show an example of the global fitting analysis done on the histograms of FRET efficiency distribution obtained during the aggregation kinetics for WT*, A53T* and A30P* aS respectively: the histograms show the relative preference for type-A or type-B oligomers for each protein, in agreement with Fig. 3B,C. Figure 5 D compares raw data of FRET efficiency histograms for each separate experiment, collected at early events in the aggregation of WT*, A53T* and A30P*, showing no major differences in the FRET efficiency distribution. Figure 5E compares the FRET efficiencies distributions obtained at the end of the lag-phase, showing that the large shift towards type-A oligomers for the majority of the A30P* samples is not due to possible bias in the fitting analysis. This experiment shows that each protein has a marked preference for a different kind of oligomers at the end of the lag-phase. Finally, Fig. 6 compares type-A oligomers (panel A) and type-B oligomers (panel B) with a statistical box-plot of the FRET efficiency values for WT* (grey), A53T* (red) and A30P* (blue) obtained from the global fitting analysis done on separate experiments for each aS variant. All the values for the centres of the Gaussian fits for each type of oligomers are reported in Table S2. For the type-A oligomers, there is no significant difference between the Gaussian centres for those formed from A53T*, A30P*, and WT* aS. However, for the type-B oligomers, there is a significant difference between the A53T* and A30P* Gaussian centres compared to that of the WT* (p < 0.05, two-samples t-test, Table S2 in Supplementary Information section). Overall, these differences in the FRET efficiency peak positions of type-B oligomers indicate that the mutation influences the oligomer structure generated from those mutants, although a more exhaustive analysis of the structures of the different oligomers with higher resolution techniques would be needed to identify the extent and nature of the structural changes of the type-B oligomers between the protein variants.

## Discussion

In our previous work, we characterized oligomers formed by aS using smFRET and obtained information about the abundance, structure and biophysical properties of the species formed during the aggregation reaction^33^,^49^,^50^. We reproducibly observed oligomers presenting two different FRET efficiency distributions^33^,^49^; these two FRET efficiency populations show different kinetics of formation and varying susceptibilities to proteinase K degradation^33^,^49^. Type-A oligomers, which exhibit a low FRET efficiency, are the first species to form. These are more susceptible to proteinase K degradation, and dissociate upon dilution into low ionic strength buffers. These subsequently convert to type-B oligomers, which are more resistant to proteinase K degradation and become incorporated into amyloid fibrils^33^,^49^. Cryo-EM, FT-IR and circular dichroism experiments on an enriched solution of oligomers exhibiting an analogous FRET efficiency revealed that they contain an extended beta-sheet structure^50^ in which the monomers are packed together tightly, therefore resulting in the higher FRET efficiency. Finally, both proteinase K resistant oligomers generated in these studies showed cell toxicity, increasing the rate of production of cytosolic ROS in neuronal primary cultures, while type-A oligomers, monomers and fibrils showed limited or no toxicity^33^,^50^.

We report here the detection and characterization of A53T*, A30P* and E46K* aS oligomers, and the comparison of them with oligomers formed from WT* aS, both in terms of their kinetics of formation, interconversion and accumulation, as well as in terms of their structural properties. We have shown that there is not an increase in the number of oligomers produced by A53T*, A30P* and E46K* proteins compared to the WT* aS (Figs 3 and 4), in contrast to previous predictions^16^,^28^,^29^. Importantly, we have found that A30P* has a much slower rate for formation of not only fibrillar species, but also for oligomers (both type-A and especially type-B), as compared with WT* and the other two pathological mutants analysed. It was previously proposed that the toxicity of A30P* may be related with a higher accumulation of oligomeric species during the aggregation of the protein, which would not be able to convert to fibrils due to the conformational constrains given by the Pro mutation^28^. We have been able to directly follow the formation of different types of oligomers and our data provide experimental evidence that there is no such increase in oligomeric concentration during the early stages of aggregation of A30P*. Instead, our results suggest that the rate limiting step for fibril-formation in this protein variant is not the progression from oligomeric to fibrillar species, but the formation and growth of type-A oligomers (half of the rate compared to WT*) and their conversion to type-B oligomers (one third of the rate compared to WT*). Once type-B oligomers are detected, fibril formation and elongation happens rapidly (Figures S2 and S4C). Together with previous studies reporting the loss of a propensity to form secondary structure for this mutant^19^,^22^,^24^ and to the well-established observation that Pro residues break the tendency of the protein to acquire beta-sheet structure^20^,^21^, our study suggests that residue 30 and/or its neighbouring residues are important for the misfolding and assembly of the protein into both type-A and type-B oligomeric species. While the protein region comprising residues 71–82 has been observed necessary for fibril formation^13^, further experimental studies together with computational predictions have shown a major influence of the N-terminal part of the protein in the first stages of the self-assembly process of the protein^53^,^54^,^55^; our experimental findings are very much in line with this idea.

The A53T* aS has been shown to have a higher tendency to acquire beta-sheet structure^19^,^24^,^26^ compared to the WT protein and we find that this higher tendency is, indeed, reflected in a higher ratio of type-B/type-A oligomers compared to the WT* (Figs 3C, S4B and 5B), suggesting that the mutation likely leads to a faster conversion from type-A to type-B oligomers^33^. The calculated conversion rate is the double of the WT* one (~5·10^−5^ s^−1^ for A53T* compared to ~2·10^−5^ s^−1^ for WT*). The observed faster rate for type-B oligomer generation is, as expected, accompanied by an earlier detection of fibrils (Figure S2).

The E46K* mutation gives rise to more complex behaviour. The mutation induces an increase in the tendency to acquire beta-sheet structure but presents an enhancement of N-terminal and C-terminal contacts^25^,^56^ leading to a more compact structure as demonstrated by a reduced gyration radius compared to the WT protein^30^,^56^. Moreover, the decrease of one net charge for the protein increases the chances for aggregation^25^,^31^; this mutation therefore interferes with many factors proposed to either enhance or inhibit the aggregation propensity of aS. Indeed, we observed a much larger variation in the lag-phase for both fibril formation (Fig. 4A) and monomer incorporation into aggregates (Fig. 4B) than for the WT* protein and the other two pathological variants. This may be a consequence of the complex effect of the E46K mutation on the conformational landscape of the protein. Solid-state NMR studies reported major differences in the structure of E46K fibrils compared to the WT protein, even far from the mutation site^57^; this may have an impact on the type of oligomers generated and explain the different behaviour of this mutant compared to the WT protein.

Although FRET efficiency values in our approach cannot be directly correlated with details of the structure of oligomers, we have observed a consistent and significant difference between the average FRET efficiency values of the type-B oligomers generated during the aggregation of the pathological mutants* and the WT* protein, which suggests that there are variations in the structure of these oligomers (Figs 5 and 6), which could further affect their potential toxic properties and/or fate in the cell. This can open to the possibility of “strains-like” phenomena induced by different aggregate morphologies of aS mutants^58^. Using FRET efficiency as a measure for oligomers compactness^33^, we can propose a less compact structure for A53T* and A30P* type-B oligomers, at least in the protein region bearing residue 90 (where the fluorescence probes were attached to). This information is complementary to the effect of these mutations on the fibril architecture, since the small soluble oligomers may have different structures from the fibrils. In fact, differences in the structure of fibrils generated from the missense mutants have been reported previously, especially for the A53T and E46K mutants, while the A30P mutation was reported to produce only minor changes^57^,^59^,^60^,^61^. Indeed, a number of studies using primarily solid-state NMR and hydrogen-deuterium exchange NMR suggest that the A30P mutation is placed in a loop at the edge of the region involved in the fibrillar structure^60^,^61^, while the A53T mutation is suggested to locate in a short beta-sheet strand causing significant structural rearrangements close to the mutation site in the fibrillar species^57^,^59^. However, a recent publication reported major differences in the structures of fibrils formed by A30P mutant and the WT aS^62^. The E46K mutation has been proposed to locate at the end of a long beta-sheet strand of the fibrillar structure, and the charge swap in this protein region has been suggested to cause major disruptions in the interaction network of this strand with the C-terminal end of the NAC region^57^. Our data may suggest that differences in the structural organization of fibrils may arise already at the oligomeric level, concretely in type-B oligomers.

## Conclusion

In this paper, we have directly followed the generation of oligomers during the lag-phase of fibril formation of WT* aS and three pathological mutants* by means of smFRET. Overall, our experimental data show that all the mutant variants produce similar concentrations and types of oligomeric species to WT protein. This is in contrast with a possible effect from aS gene duplication and triplication, which is likely to increase the number of oligomers produced. In addition, we found that one amino acid substitution in aS is able to induce significant differences in the kinetics of formation of oligomers, towards a preference for the accumulation of different types of oligomers depending on the type and position of the mutation, for type-A in the case of A30P* and for type-B in the case of A53T*, while in other cases a complex mixture of species is generated (E46K*). This is probably due to a differences in the conversion rates between WT* and mutants* aS. Furthermore, all these mutations seem to induce significant differences in the structure of the type-B oligomers, according to the analysis of their FRET efficiency values, and these structural variations at the oligomeric level among the protein variants are likely to be transferred into the structure of mature fibrils.

## Methods

### Single-molecule Fluorescence Kinetics

A solution of A90C aS labelled via maleimide chemistry with AlexaFluor 488 (Life Technologies) was mixed with AlexaFluor 594 labelled aS and diluted to 70 μM with equimolar concentration of donor and acceptor species in 25 mM Tris-HCl, pH 7.4, 100 mM NaCl, and put at 37 °C upon shaking. Buffer was freshly prepared and filtered with 0.02 μm filters (Anotop, Whatman). Regular time-points are taken and centrifuged for at least 10’ at 14,000 rpm in a table-top centrifuge. 2 μl of surnatant were taken for smFRET measurement and diluted 250000 times in the same buffer used for the aggregation. The aggregation was stopped approximately at two time-points after the formation of a pellet upon sample centrifugation. The fluorescent label on aS causes a small delay in fibril formation but otherwise there was no detectable changes in its aggregation, as verified by TEM on the pellet formed upon centrifugation (see supplementary Information section, Figure S3). The data presented are the average of 3 experiments for WT*, 5 experiments for A53T*, 6 experiments for A30P* and 7 experiments for E46K* (* denotes labelling). We noted the presence of preformed oligomeric species for A30P* before the aggregation reaction started, although these species were observed to dissociate after the aggregation reaction was initiated, suggesting they do not initiate the aggregation process leading to fibril formation (this behaviour was also observed occasionally for the WT* protein and it was previously reported^33^). For the data in Fig. 6, data from incomplete kinetic analysis were included to obtain FRET efficiency for WT* aS and mutants*, until the collection of 8 distributions for each sample. Details for mutants cloning, expression and labelling are reported in the Supplementary Information section.

### Single Molecule Fluorescence instrument set up and measures

The instrumentation for single molecule FRET measurement has been reported in detail^48^ with some modifications. A 488 nm (Spectra Physics Cyan CDRH) laser beam is directed to the back port of a Nikon Eclipse Ti-U microscope, and reflected by a dichroic mirror through an objective (Apochromat 60×, NA 1.40, Nikon) to be carefully focused into the centre of a microfluidic channel 10 μm above the coverslip surface. Emission bursts were collected by the same objective and directed onto a 50 μm pinhole (Melles Griot). A set of dichroics separated the emission from the two different fluorophores that were focused and directed onto Avalanche Photodiode (APD) detectors. Dark count rates for the APDs did not exceed 100 counts/s. Signals detected from the APDs were recorded and binned in time by a custom-programmed field programmable gate array (FPGA) (Colexica). The cross-talk from donor into acceptor channel was found to be 9%, while negligible from acceptor into donor channel. The laser power was 2 mW for 488 nm excitation. For all of the single-molecule experiments, data were collected at 20 °C running diluted samples from aggregation kinetics at 2 cm/s through a 100 μm microfluidic channel (please see Supplementary Information for details in the fabrication of the devices). Flow was achieved using a syringe pump (PHD2000, Harvard Apparatus) to apply a negative pressure at the outlet, while the samples were loaded into a gel tip acting as a reservoir at the inlet. Fluorescence counts were combined into 50 μs time-bins in both channels (expected residence time of molecules). Typically, 80 frames of 100,000 bins were collected for a total measurement time of 400 s per sample. Parameters have been selected to allow the best detection efficiency as previously described^49^.

### Data analysis

Details from the data analysis procedure are reported in Horrocks *et al.*^49^. A procedure written in Igor Pro (Wavemetrics) was used to read output data from the FPGA card, which were two arrays of 32 bit signed integers, corresponding to time-binned photon bursts in the two emission channels. Any time-bins in which there is concurrently a greater intensity than an pre-set threshold in both the donor and acceptor channel (AND criterion) are assigned to oligomeric; the donor channel signals which do not fit this criterion, but have intensities greater than the donor threshold are kept as deriving from monomeric aS. The cross-talk and the auto-fluorescence values (which depend on the conditions used) are subtracted from the data collected:


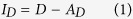



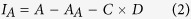


where *I*_*D*_ is the modified intensity in the donor channel, *D* is the original intensity and *A*_*D*_ is the auto-fluorescence in the donor channel, *I*_*A*_ is the modified intensity in the acceptor channel, *A* is the original intensity in the acceptor channel, *A*_*A*_ the acceptor channel auto-fluorescence, and *C* the cross-talk from donor to acceptor channel.

Approximate size of the oligomer (*Size*) and FRET efficiency (*E*) are calculated for each burst assigned to oligomers according to the following equations:


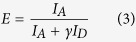



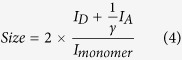


where *γ* is the experimentally determined gamma factor, corresponding to the relative detection efficiencies of the two dyes by the instrumentation and their quantum yields, and *I*_*monomer*_ is the average monomer brightness, calculated from the average of the non-coincident donor channel bursts intensities. Equation 4 gives an approximate estimate of the number of monomeric aS present in each oligomer. Since only donor-labelled aS in the oligomer can be directly excited but it can then emit in the donor or acceptor channel, the total burst fluorescence intensity (as sum of donor and acceptor intensity) will be proportional to the number of donor fluorophores in the oligomer (the γ-factor here corrects for the difference in detection efficiencies between the donor and acceptor photons). To estimate the oligomer size it is assumed that there are, on average, equal numbers of donors and acceptors in an oligomer, therefore, the total intensity is multiplied by 2 in Equation 4. To estimate the oligomer size (in monomeric units), the sum is then divided by the mean monomer brightness. Fluorescence lifetime measures showed no significant quenching of the donor and acceptor molecules in the oligomers (data not shown). Due to the stochastic nature of excitation and emission and to the possible different paths that the fluorophore can take through the confocal volume, size estimation based on intensity is only approximate. In these experiments any large species (>150 monomers) or species occupying more than one time-bin were assumed to be fibrils and not included in the analysis (evidence for this is provided in Horrocks *et al.*, 2015)^49^.

Oligomers have been divided into three groups depending on their apparent size as already published^33^,^49^. Small oligomers correspond to apparent dimers, the remaining of the oligomers are englobing 3 to 150 units of monomer brightness intensities. The division into type-A and type-B oligomers have been made plotting data obtained from oligomers in dependence on their FRET efficiency and relative abundance among the species; global fitting analysis has been performed to fit data into one or two Gaussian distributions. In the case of two distributions, they have been named type-A and type-B oligomers based on the absolute value of FRET efficiency of the identified centre of the Gaussian curve. These criterions have been applied to produce data shown in Figs 3,5 and 6 and S4, S5. Single-molecule data are normalized on the total number of events (identified as monomers or oligomers) and the relative amount of monomer left in solution (Figure S2).

## Additional Information

**How to cite this article**: Tosatto, L. *et al.* Single-molecule FRET studies on alpha-synuclein oligomerization of Parkinson’s disease genetically related mutants. *Sci. Rep.* **5**, 16696; doi: 10.1038/srep16696 (2015).

## Supplementary Material

Supplementary Information

## Figures and Tables

**Figure 1:**
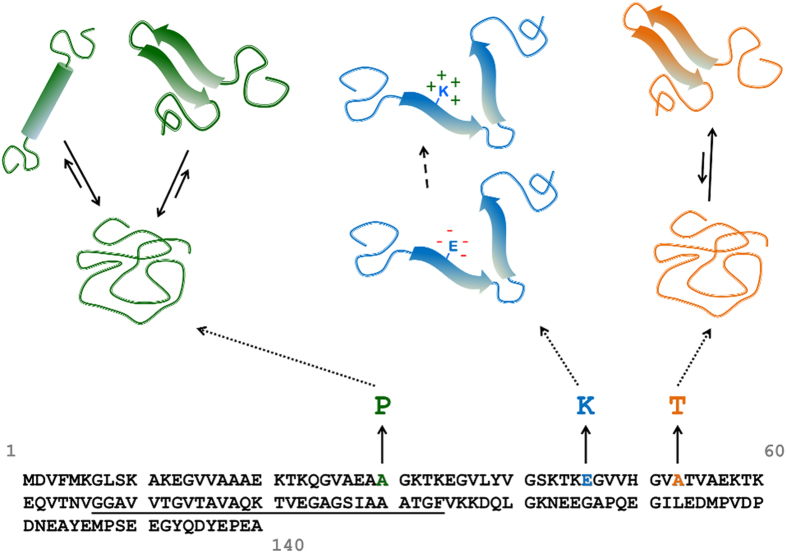
Primary structure of WT aS with sites of pathogenic missense mutations highlighted.
