# Supplementary material for: Single-molecule FRET studies on alpha-synuclein oligomerization of Parkinson’s disease genetically related mutants
Source: Sci Rep. 2015 Nov 19;5:16696. doi: 10.1038/srep16696 (PMC4652217; doi:10.1038/srep16696)
Supplement: Supplementary Information [file srep16696-s1.pdf]

# Single-molecule FRET studies on alpha-synuclein oligomerization of Parkinson's disease genetically related mutants

Laura Tosatto[a,b,1], Mathew H. Horrocks[a,1], Alexander J. Dear[a], Tuomas P. J. Knowles[a], Mauro Dalla Serra[b], Nunilo Cremades[a,c], Christopher M. Dobson[a], David Klenerman[a,*]

a) Department of Chemistry, University of Cambridge, Lensfield Road, CB2 1EW Cambridge, UK
b) Istituto di Biofisica, CNR, U.O. Trento, via alla Cascata 56/C, 38123 Povo (TN), Italy
c) Institute for Biocomputation and Physics of Complex Systems (BIFI), Universidad de Zaragoza, Mariano Esquillor, Edificio I + D, 50018 Zaragoza, Spain.

1) These authors contributed equally to this work

* To whom correspondence should be addressed. E-mail: dk10012@cam.ac.uk

## SUPPLEMENTARY INFORMATION

**Abbreviations**

PD, Parkinson's disease; aS, alpha-synuclein; WT, wild-type; smFRET, single molecule Förster Resonance Energy Transfer.

**Supplementary Methods**

*Materials*. Molecular biology enzymes were purchased from Fermentas (Thermo Fisher). Media for protein expression were purchased from Novagen (Merck). Fluorescent dyes have been purchased from Life Technologies. All other chemicals were purchased from Sigma Aldrich.

*Protein cloning.* pT7-7 plasmids containing genes for WT aS, A53T, A30P, E46K and A90C were used. The Alanine in position 90 was modified to a Cysteine by mutagenic PCR using as forward primer the sequence 5'-GCAGGGAGCATTGCATGTGCCACTGGCTTTGTC and reverse 5'-GACAAAGCCAGTGGCACATGCAATGCTCCCTGC (Eurofins MWG Operon). PCR is set for 25 cycles of 30" at 95°C, 1' at 55°C, 4' at 68°C. The elongation reaction was completed at the end of the cycle with 7' at 72°C. The PCR reaction mix was digested with DnpI for 1 h at 37°C and cleaned after the reaction with a PCR cleaning kit from Qiagen. Xl-1 Blue cells (Stratagene) were transformed and left to grow overnight in LB agar plates supplemented with 100 µg/ml of ampicillin. Colonies were screened by sequencing.

*Protein expression and purification.* BL21(DE3) Gold cells (Stratagene) were transformed with WT aS, A53T, A30P, E46K and Cysteine mutants A90C, A53T-A90C, A30P-A90C and E46K-A90C. Starters were diluted into Overnight Express™ Instant TB Medium (Novagen) supplemented with 1% glycerol and left to grow for 16-18 h at 30°C. After that the cells were harvested and the protein was purified as previously published[1].

*Protein labelling*. Cysteine variants of WT aS and mutants (A90C, A53T-A90C, A30P-A90C and E46K-A90C) were labelled as reported previously[1]. Life Technologies Alexa Fluor 488

$C_5$ maleimide and Alexa Fluor 594 $C_5$ maleimide were used in these reactions. The labelled protein was purified from unreacted dye using the method reported in Cremades et al., 2012[1]. The reaction yield was checked by mass spectrometry, and proteins labelled less than the 90% were not used in experiments. In order to avoid confusion with unlabelled proteins used in other experiments, labelled A90C, A53T-A90C, A30P-A90C and E46K-A90C will be defined as WT*, A53T*, A30P* and E46K*.

*Protein aggregation for Thioflavin T assay*. The A53T and A30P pathological aS variants have been reported to have a shorter lag-phase and a longer lag-phase, respectively, than the WT protein for their aggregation reaction *in vitro* (A53T>WT>A30P)[2]. In order to confirm the different aggregation propensities of the aS mutants are maintained upon the aggregation conditions used for smFRET, a Thioflavin T assay was performed using unlabelled protein, as the fluorescent labels used for the single-molecule fluorescence experiments fluoresce at similar wavelengths to Thioflavin T. WT aS or the Parkinson's disease mutants were prepared from gel filtration purified monomers fractions flash frozen in liquid nitrogen. Experiments were conducted in triplicate. For each experiments, 600 µL of 70 uM protein in 25 mM Tris-HCl pH 7.4 and 100 mM NaCl were incubated in the dark at 37°C upon 200 rpm orbital shaking (25 mm shaking diameter). Each reaction was supplemented with 0.01 % $NaN_3$ to prevent bacterial growth. At defined time-points, 10 µl of aggregation mixture were collected and stored at 4°C. Stock solutions of Thioflavin T were prepared in ethanol and the concentration was checked by UV-VIS absorbance using the extinction coefficient of 26,620 $M^{-1}$ $cm^{-1}$ at 416 nm. The stock was diluted to 20 µM in the same buffer as the aggregation mixture just before fluorescent measurements. 90 µl of 20 µM Thioflavin T was added to the aggregation aliquot; the mixture was vortexed and then incubated for 30' at room temperature. Measurements were run in a 60 µL volume cuvette with 3 optical quartz windows (Hellma). Fluorescence intensity measurements were recorded with a Cary Eclipse (Varian) accumulating 3 emission spectra from 460 nm to 600 nm. The excitation wavelength was set at 446 nm and the excitation and emission slits were set at 5 nm each. Measurements were taken at 25°C. Curve fitting has been calculated with Origin software considering parameters for Boltzmann equation yielding the least chi squared value. WT aS had an intermediate value for the aggregation lag-phase compared to A53T and A30P (Figure S1). This indicates that the aggregation propensity is A53T>WT>A30P, in agreement with previously reported works[2]. When compared the kinetics of aggregation for the unlabelled

and fluorescently labelled protein variants, we found very similar behaviours and the same relation and trend between the characteristic time for the lag-phase of aggregation for the pathological and WT protein, although the incorporation of the probe is consistently increasing slightly the lag-phase of all the protein variants[1].

*Time-scale of aggregation selection criteria.* Before starting the smFRET experiment, it is important to define the time scale of the aggregation reaction, both to check that the aggregation propensities for the mutants is not altered by the labelling and to identify at which point the reduction in monomer concentration can potentially affect the rate of oligomer formation due to the lack of monomeric protein. Since the Thioflavin-T fluorescence cannot be measured with fibrils formed from fluorescently tagged proteins, we followed fibril-formation on the labelled protein by incubating an equimolar mixture of either AlexaFluor 488 or AlexaFluor 594 labelled aS, taking regular time-points, and centrifuging and measuring the absorbance of the supernatant at 495 nm and 594 nm (corresponding to the maximum extinction coefficient of the two dyes). The monomeric fraction in the aggregation solution was measured by absorbance of AlexaFluor 488 and AlexaFluor594 labelled proteins using a Nanodrop (Thermo Fisher). Both labelled proteins were incorporated with the same rate into fibrils (SI Figure S6). The monomer fractions reported are calculated as an average between the absorbance of AlexaFluor488 and AlexaFluor594 labelled proteins, considering values obtained from 3 to 7 aggregations. This gives a measure of the fraction of protein remaining in solution, and Figure S2 shows results for labelled WT (WT*, grey signals), A53T (A53T*, red signals) and A30P (A30P*, blue signals). The decrease in absorbance is slow until a visible pellet is observed (arrows in Figure S2A), at which point it decreased rapidly, indicating that monomers are also able to directly add to fibrils[3]. The experiment was stopped after the deposition of a pellet upon centrifugation but generally two smFRET time-points are analysed after the detection of the protein pellet. There is a small delay compared to the unlabelled protein Thioflavin T assay in Figure S1, but the ability of the protein to aggregate is not affected by the fluorescent tag, as checked by TEM on the pellet formed upon centrifugation (Figure S3). We then divided the time window for the analysis in two parts: "lag-phase", where no fibrils are present, corresponding to the first days of reaction for WT* and A53T* and the first 48 hours for A30P* (5 time-points for each variant) and "end of the lag-phase", corresponding to the three time-points where the fibrils pellet starts to be visible (30 ± 2 hours, for A53T* at 26 ± 2 hours and for A30P* at 72 ± 16 hours).

*Transmission Electron Microscope imaging*. For TEM imaging, WT* labelled with AlexaFluor488 only, or AlexaFluor594 or the equimolar mixture both, were incubated under aggregating conditions. 10 µl of each sample were taken after 48 hours of incubation and applied onto carbon-coated 400-mesh copper grids (Agar Scientific) for 5 min, and then washed with distilled water. Negative staining was carried out by using 2 % (w/v) uranyl acetate. TEM images were acquired using Tecnai G2 microscope (13218, EDAX, AMETEK) operating at an excitation voltage of 200 kV.

*Production of microfluidic devices*. Microfluidic devices have been prepared as previously described[4]. The devices were configured with AutoCAD 2007 design software (Autodesk), and a negative mask was printed (Circuit graphics). SU-8 2025 photoresist (MicroChem) was homogeneously distributed on a silicon wafer (∅ 76.2 mm, Compart Technology Ltd.) surface via spin-coating (800 rpm for 5 s and then ramped to 3000 rpm at an acceleration of 300 rpm/s for 60 s). The final film thickness was 25 µm, measured by profilometry (DekTak 150). After spinning, the wafer was incubated 1 min at 65°C, then 3 min at 95°C and finally 1 min at 65°C, and then exposed to UV light through the mask on a mask aligner (MJB4, SUSS Microtec). After post-baking and development, the master was incubated for 1 min at 170°C. PDMS and curing agent (Sylgard 184, Dow Corning) in a 10/1 w/w ratio were poured over the master, degassed and baked at 75°C at least 2 hours. The devices were separated from the master and cut, using a biopsy punch to introduce access holes for the inlet tubes. The devices were then exposed to oxygen plasma for 7 s (DienerFemto plasma asher), sealed to a glass microscope slide and baked overnight at 75°C.

*Calculation of the gamma factor ($\gamma$) and association quotient (Q) used for data analysis.* The correction factor ($\gamma$) was calculated using a solution of labelled AlexaFluor488 and AlexaFluor594 aS at concentrations enabling the dyes to yield the same absorption at 488 nm (Varian Eclipse Spectrophotometer). The solution was then measured in bulk on the single-molecule instrument, giving $\gamma = 1.01$ according to:

$$\gamma = \frac{I_D}{I_A} \qquad \text{(equation S1)}$$

where $I_D$ and $I_A$ are the intensities in the donor and acceptor channels[5].

Q is the association quotient used to report the ratio of the rate of coincident events to the sum of the rate of all events in the donor and acceptor channels, subtracting the chance coincident events as indicated[6]:

$$Q = \frac{(C-E)}{A+B-(C-E))} \qquad \text{(equation S2)}$$

where $A$ and $B$ are the event rates ($s^{-1}$) in donor and acceptor channels and $C$ the observed rate of coincident events, and the estimated rate at which coincident events occur by chance ($E$) is

$$E = AB\tau \qquad \text{(equation S3)}$$

where $\tau$ is the interval time in seconds.

*Fitting data to model equation.* A general kinetic model[7] can be used to fit the kinetics of the early stages of amyloid fibril formation observed in aS smFRET data. Some assertions are considered in the design of this model:

- two classes of oligomeric intermediates (type-A and type-B) are considered explicitly; while type-B oligomers are partly structured and present characteristics closer to amyloid fibrils, type-A oligomers are less stable compared to type-B and structurally distinct from the former species[1].
- Both type-A and type-B oligomers can grow through monomer addition, and undergo the inverse phenomenon of monomer dissociation.
- There is no direct nucleation of type-B structures from monomer.
- Type-A oligomers of a given number of monomers can convert into type-B structures.

The time evolution describing the number concentration, growth and time evolution of type-A and B oligomers can be written in terms of primary nucleation, elongation and interconversion between species. The equations for number concentrations of oligomer types A and B are obtained summing over all possible oligomer lengths. We restrict the application of the equations to the system only to the period before the bulk lag-time for fibril formation, $\tau_{lag}$, as the concentration is approximately constant at its initial value due to the fact that only few monomers have been engaged into aggregates. In this regime, many processes as reverse conversion from type-B to A depolymerisation, accounted for in the master equation, do not have a significant effect on the number-concentrations of oligomers and can be ignored. Under these conditions, the number concentration equations can be written as:

$$\frac{dQ}{dt} = k_n m_{tot}^{n_c} - k_c Q \qquad \text{(equation S4)}$$

$$\frac{dP}{dt} = k_c Q \qquad \text{(equation S5)}$$

where $Q$ is the number concentration of oligomers of type-A, $k_n$ type-A oligomer formation rate, $m_{tot}$ monomer concentration, $k_c$ type-A oligomers conversion rate and $P$ the concentration of type-B oligomers. These can then be solved in closed form, giving the solutions for the number concentrations as follows:

$$Q = \frac{k_n}{k_c} m_{tot}^{n_c}(1 - e^{-k_c t}) \qquad \text{(equation S6)}$$

$$P = \frac{k_n}{k_c} m_{tot}^{n_c}(k_c y + e^{-k_c t} + 1) \qquad \text{(equation S7)}$$

where non-seeded initial conditions, $Q(0) = P(0) = 0$, have been used. Results permit to determine constants $k_n'$ $( = k_n m_{tot}^{nc-1})$ and $k_c$ from the number concentrations of type-A and type-B oligomers in the restricted time period consider (data have been fitted till 30 hours for WT*, 26 hours for A53T* and 56 hours for A30P*). The experimental data was fitted to Equations S6 and S7 up to the lag-time and fits were obtained using the two kinetic parameters $k_n'$ and $k_c$. $\tau_{lag}$ has been identified through the measure of bulk soluble monomer concentration shown in Figure S2. In order to fit data, data expressed in “fraction of events” have been multiplied by initial monomer concentration (70 µM) to provide an estimation of oligomers molarity (Figure S4). The results of the fitting are reported in Figure S4 (continuous lines); curves were obtained setting $k_n$’ and $k_c$ as $5\cdot10^{-8}$ $s^{-1}$ and $2\cdot10^{-5}$ $s^{-1}$ for WT* aS, $4\cdot10^{-8}$ $s^{-1}$ and $5\cdot10^{-5}$ $s^{-1}$ for A53T* and $2\cdot10^{-8}$ $s^{-1}$ and $6\cdot10^{-6}$ $s^{-1}$ for A30P*. These values are consistent with literature[1] (and Horrocks et al., 2015, submitted). Stronger accordance was not awaited due to the noise inherent to smFRET data.

**Supplementary Table S1.** Percentage of oligomers (=fraction of events·100) deducted from global fitting analysis for apparent dimers, type-A and B oligomers for WT*, A53T* and A30P*. The error reported is SE. Time-points considered for histograms in Figure 3 B and C are highlighted in blue (lag-phase) or grey (end of lag-phase), respectively.

| Time (h) | Dimers | Type-A | Type-B | Total |
|---|---|---|---|---|
| WT | | | | |
| 0 | 0.028±0.005 | 0.044±0.025 | 0.009±0.005 | 0.081±0.035 |
| 2 | 0.045±0.015 | 0.076±0.029 | 0.048±0.033 | 0.170±0.077 |
| 4 | 0.074±0.007 | 0.146±0.052 | 0.017±0.013 | 0.237±0.072 |
| 6 | 0.058±0.003 | 0.115±0.042 | 0.062±0.020 | 0.235±0.065 |
| 8 | 0.080±0.014 | 0.187±0.085 | 0.053±0.035 | 0.320±0.135 |
| 10 | 0.117±0.048 | 0.292±0.189 | 0.100±0.045 | 0.509±0.281 |
| 24 | 0.171±0.082 | 0.306±0.134 | 0.134±0.082 | 0.611±0.298 |
| 26 | 0.092±0.008 | 0.127±0.013 | 0.293±0.074 | 0.512±0.095 |
| 28 | 0.115±0.017 | 0.182±0.028 | 0.136±0.065 | 0.433±0.110 |
| 30 | 0.161±0.064 | 0.376±0.122 | 0.352±0.264 | 0.889±0.450 |
| 32 | 0.159±0.026 | 0.194±0.053 | 0.314±0.115 | 0.667±0.194 |
| 48 | 0.285±0.026 | 0.929±0.291 | 0.387±0.138 | 1.601±0.455 |
| A53T | | | | |
| 0 | 0.087±0.011 | 0.065±0.008 | 0.063±0.020 | 0.215±0.039 |
| 2 | 0.088±0.014 | 0.097±0.038 | 0.027±0.007 | 0.212±0.060 |
| 4 | 0.132±0.015 | 0.080±0.015 | 0.115±0.043 | 0.327±0.074 |
| 6 | 0.148±0.058 | 0.095±0.024 | 0.029±0.019 | 0.272±0.101 |
| 8 | 0.082±0.003 | 0.081±0.008 | 0.023±0.005 | 0.186±0.016 |
| 10 | 0.079±0.014 | 0.082±0.024 | 0.028±0.018 | 0.189±0.056 |
| 12 | 0.111±0.023 | 0.051±0.012 | 0.061±0.014 | 0.223±0.049 |
| 24 | 0.193±0.014 | 0.006±0.004 | 0.365±0.048 | 0.564±0.066 |
| 26 | 0.227±0.034 | 0.101±0.049 | 0.312±0.066 | 0.640±0.148 |
| 28 | 0.301±0.076 | 0.093±0.020 | 0.128±0.042 | 0.522±0.138 |
| 30 | 0.242±0.150 | 0.012±0.012 | 0.198±0.181 | 0.452±0.343 |
| A30P | | | | |
| 0 | 0.241±0.050 | 0.207±0.100 | 0.144±0.042 | 0.592±0.192 |
| 8 | 0.209±0.066 | 0.168±0.096 | 0.061±0.015 | 0.438±0.177 |
| 24 | 0.192±0.084 | 0.105±0.036 | 0.058±0.014 | 0.355±0.134 |
| 32 | 0.120±0.012 | 0.104±0.051 | 0.065±0.015 | 0.289±0.077 |
| 48 | 0.216±0.092 | 0.093±0.036 | 0.138±0.021 | 0.447±0.149 |
| 56 | 0.232±0.139 | 0.285±0.171 | 0.100±0.048 | 0.617±0.358 |
| 72 | 0.368±0.086 | 0.491±0.216 | 0.327±0.090 | 1.186±0.392 |
| 80 | 0.225±0.076 | 0.550±0.282 | 0.424±0.215 | 1.199±0.573 |
| 96 | 0.315±0.100 | 0.750±0.427 | 0.163±0.036 | 1.228±0.563 |

**Supplementary Table S2**. List of FRET efficiency values obtained from type-A and type-B oligomers using global fit analysis. In the t-test columns, two-samples independent t-test has been run comparing each mutant with values collected for the wild type protein. Data are normally distributed according to the Shapiro-Wilk test.

| Protein | Type-A | | | Type-B | | |
|---|---|---|---|---|---|---|
| | Values | Mean ± sd | t-test | Values | Mean ± sd | t-test |
| WT* | 0.34 | 0.35 ± 0.06 | / | 0.55 | 0.59 ± 0.02 | / |
| | 0.45 | | | 0.62 | | |
| | 0.44 | | | 0.61 | | |
| | 0.34 | | | 0.59 | | |
| | 0.31 | | | 0.59 | | |
| | 0.32 | | | 0.59 | | |
| | 0.33 | | | 0.61 | | |
| | 0.28 | | | 0.56 | | |
| A53T* | 0.27 | 0.33 ± 0.06 | n.s. | 0.50 | 0.53 ± 0.05 | P < 0.05 (P=0.010) |
| | 0.26 | | | 0.48 | | |
| | 0.30 | | | 0.49 | | |
| | 0.38 | | | 0.56 | | |
| | 0.38 | | | 0.51 | | |
| | 0.44 | | | 0.58 | | |
| | 0.29 | | | 0.52 | | |
| | 0.34 | | | 0.62 | | |
| A30P* | 0.35 | 0.35 ± 0.08 | n.s. | 0.47 | 0.49 ± 0.08 | P < 0.05 (P=0.003) |
| | 0.33 | | | 0.46 | | |
| | 0.23 | | | 0.40 | | |
| | 0.25 | | | 0.38 | | |
| | 0.44 | | | 0.56 | | |
| | 0.42 | | | 0.53 | | |
| | 0.44 | | | 0.59 | | |
| | 0.33 | | | 0.53 | | |

**Supplementary Information References**


1. Cremades, N. *et al.* Direct Observation of the Interconversion of Normal and Toxic Forms of α-Synuclein. *Cell* **149,** 1048–1059 (2012).
2. Li, J., Uversky, V. N. & Fink, A. L. Effect of familial Parkinson's disease point mutations A30P and A53T on the structural properties, aggregation, and fibrillation of human alpha-synuclein. *Biochemistry (Mosc.)* **40,** 11604–11613 (2001).
3. Buell, A. K. *et al.* Solution conditions determine the relative importance of nucleation and growth processes in α-synuclein aggregation. *Proc. Natl. Acad. Sci. U. S. A.* **111,** 7671–7676 (2014).
4. Horrocks, M. H. *et al.* Single molecule fluorescence under conditions of fast flow. *Anal. Chem.* **84,** 179–185 (2012).
5. Ye, Y. *et al.* Ubiquitin chain conformation regulates recognition and activity of interacting proteins. *Nature* **492,** 266–270 (2012).
6. Orte, A., Clarke, R., Balasubramanian, S. & Klenerman, D. Determination of the fraction and stoichiometry of femtomolar levels of biomolecular complexes in an excess of monomer using single-molecule, two-color coincidence detection. *Anal. Chem.* **78,** 7707–7715 (2006).
7. Garcia, G. A., Cohen, S. I. A., Dobson, C. M. & Knowles, T. P. J. Nucleation-conversion-polymerization reactions of biological macromolecules with prenucleation clusters. *Phys. Rev. E Stat. Nonlin. Soft Matter Phys.* **89,** 032712 (2014).

## SUPPLEMENTARY FIGURES

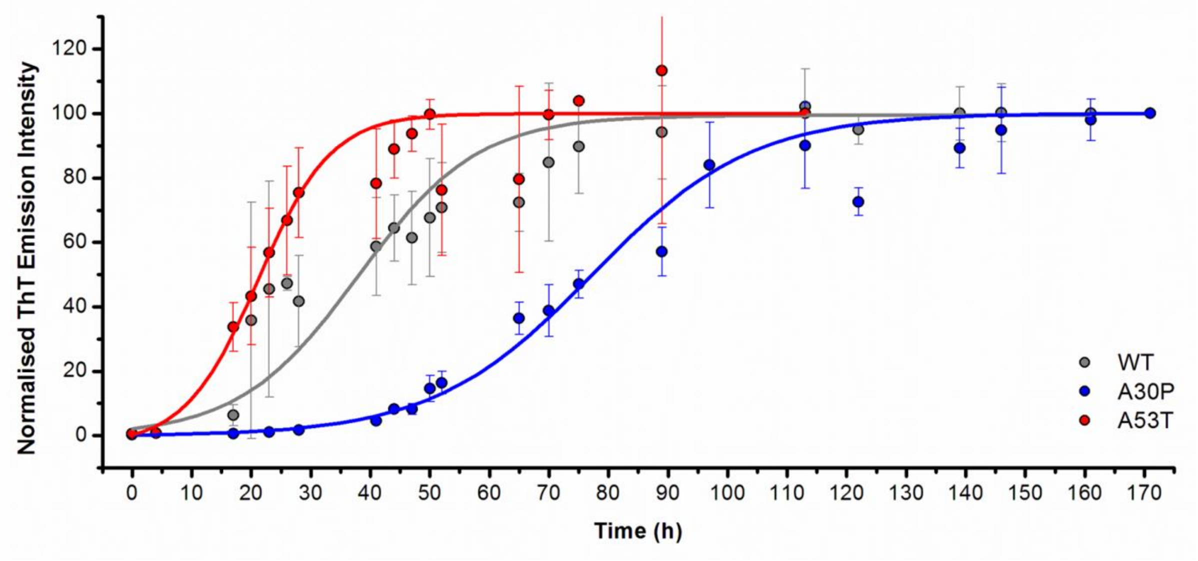


**Supplementary Figure S1**. Thioflavin T assay for WT aS (grey) and A53T (red), A30P (blue) mutants. Reported results correspond to the mean of three different experiments. Error bars are SEM; signals have been normalized to the plateau level.

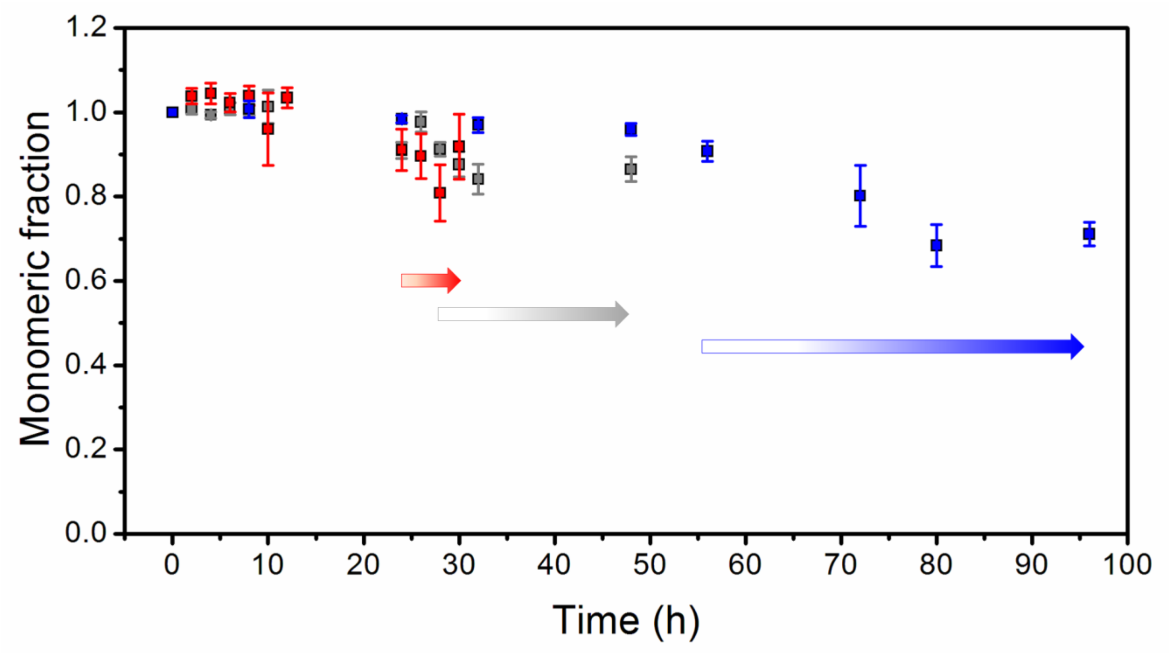


**Supplementary Figure S2**. (A) Monomer concentration decay for WT* (grey; n=3), A53T* (red; n=5) and A30P* (blue; n=6) from the mean absorbance of donor and acceptor labelled proteins within the supernatant after centrifugation. The grey, red and blue arrows indicate the presence of fibrils in solution upon centrifugation of WT*, A53T* and A30P* samples, respectively.

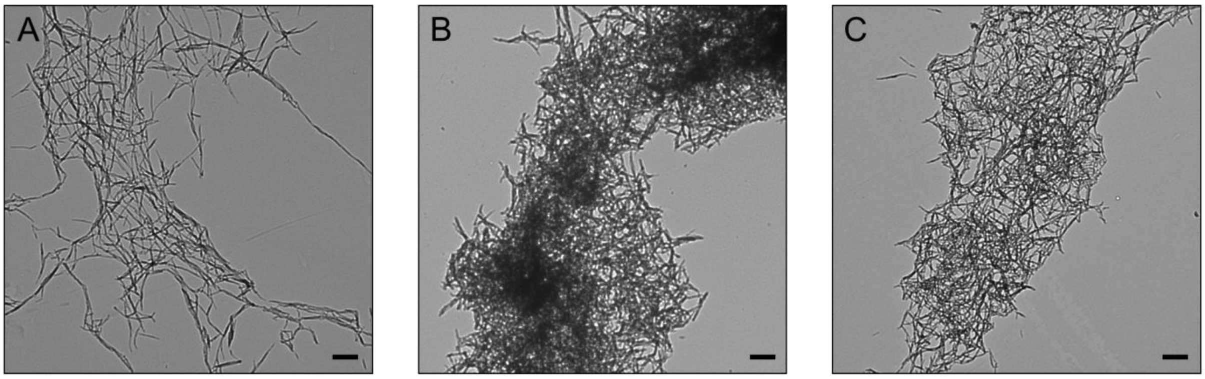


**Supplementary Figure S3**. (A) TEM images on WT* aS labelled with AlexaFluor-488 only. (B) TEM images on WT* aS labelled with AlexaFluor-488 and AlexaFluor-594. (C) TEM images on WT* aS labelled with AlexaFluor-594 only. The bar corresponds to 500 nm.

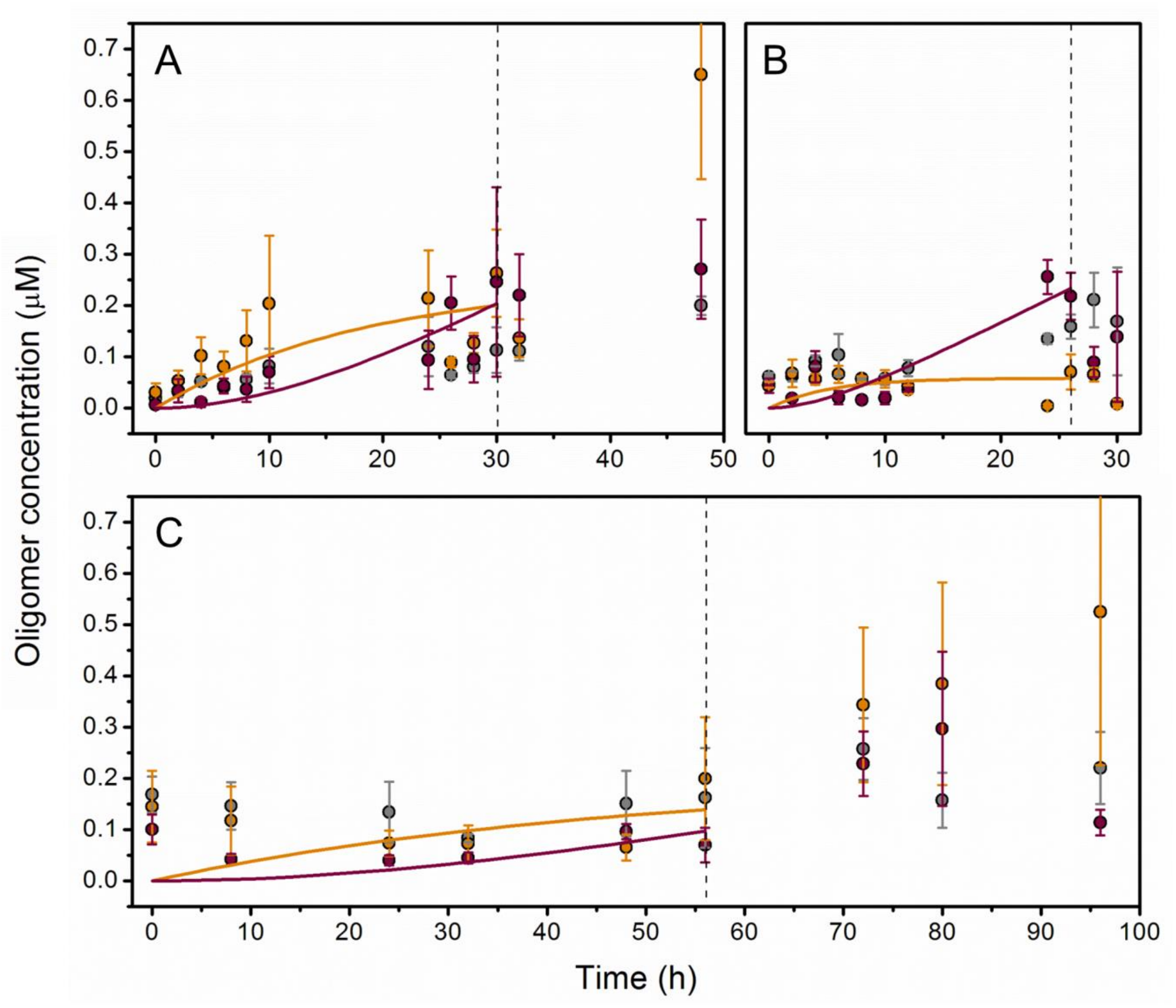


**Supplementary Figure S4**. Single-molecule fluorescence kinetics for the detection of WT* (panel A), A53T* (panel B) and A30P* (panel C) oligomers. Grey, orange and purple traces are indicative for the estimation of apparent dimers, type-A oligomers, and type-B oligomers, respectively. Continuous lines indicate the results of the fitting of smFRET into the model for type-A (orange) and type-B (purple) oligomer formation and conversion. Error bars reported are SEM. Dashed lines report the point in which soluble monomer concentration decreases more than 90% and fibril detection occurs; this results in the addition of other phenomena like the formation of large fibrillar species and dissociation of type-A oligomers which become significant and may affect the quality of the fitting.

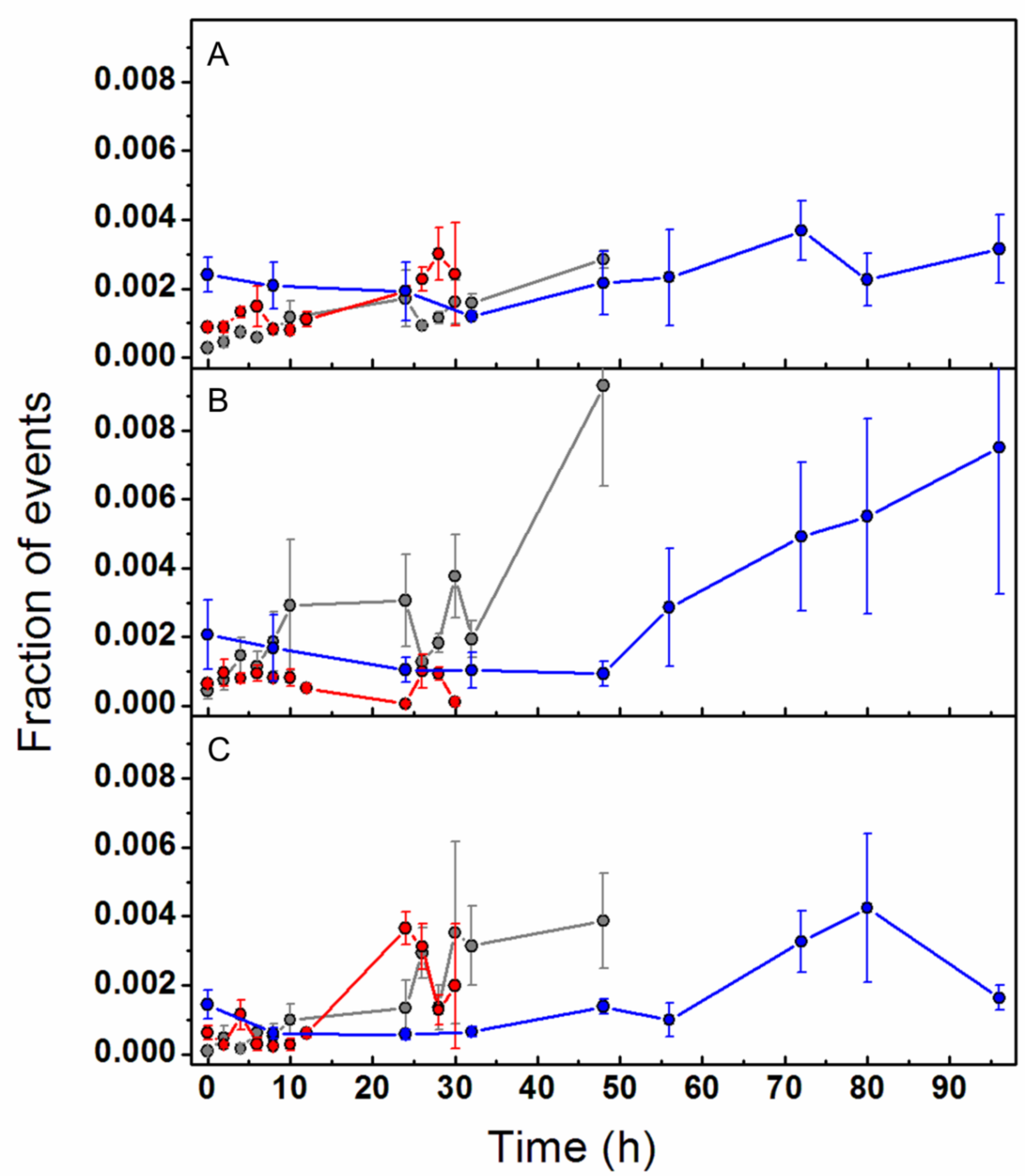


**Supplementary Figure S5.** For a better comparison of kinetic of formation of different species by WT*, A53T* and A30P*, data are re-plotted from Figure S4, reporting the formation of apparent dimers, type-A oligomers and type-B oligomers, respectively. (A) Fraction of events reporting the kinetic of formation of apparent dimers for WT* (grey), A53T* (red) and A30P* (blue). (B) Fraction of events reporting the formation of type-A oligomers for WT* (grey), A53T* (red) and A30P* (blue). (C) Fraction of events reporting the formation of type-B oligomers for WT* (grey), A53T* (red) and A30P* (blue). Error bars reported are SEM.

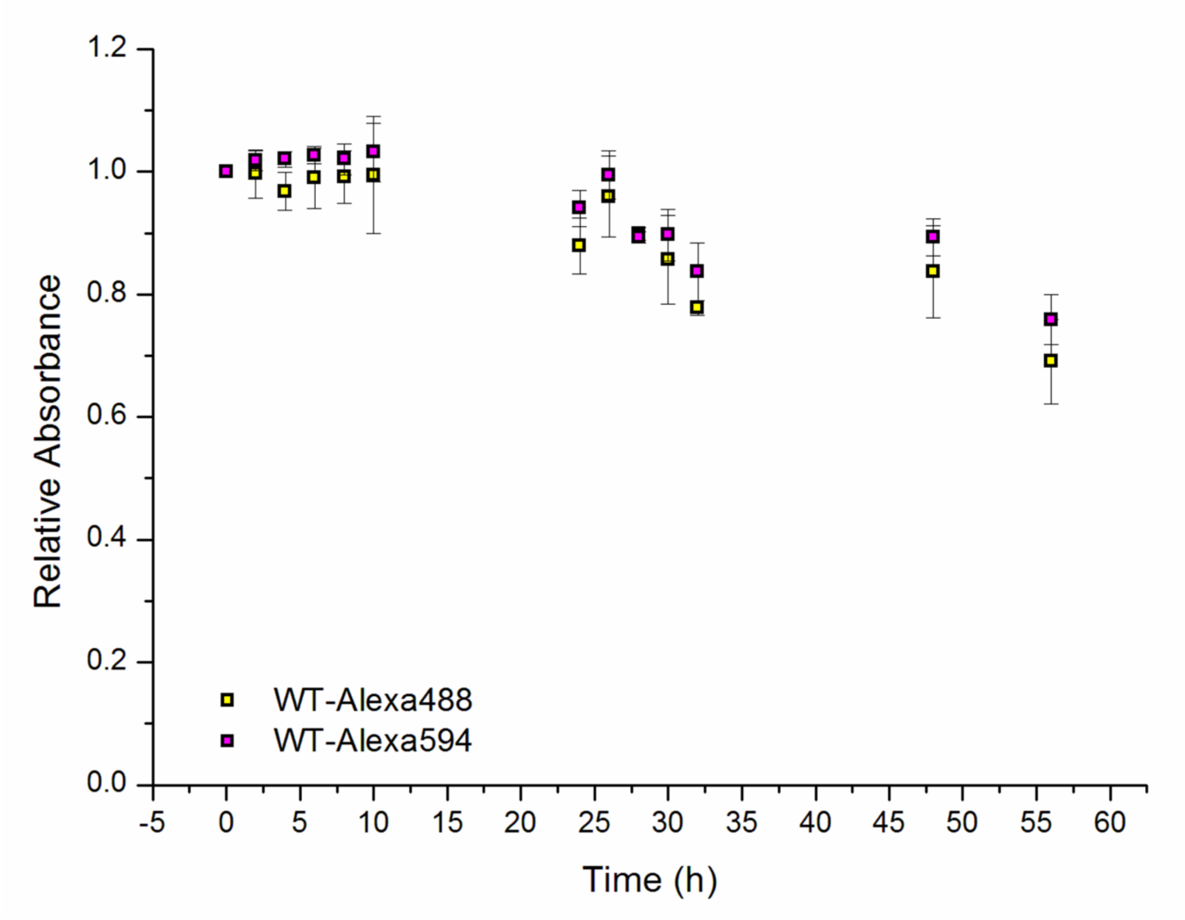


**Supplementary Figure S6**. Relative monomer concentration of WT aS labelled with AlexaFluor 488 or AlexaFluor 594 during aggregation kinetics showing the same incorporation rate of the two differently labelled proteins in the fibrils. Error bars are SD of three independent experiments.
